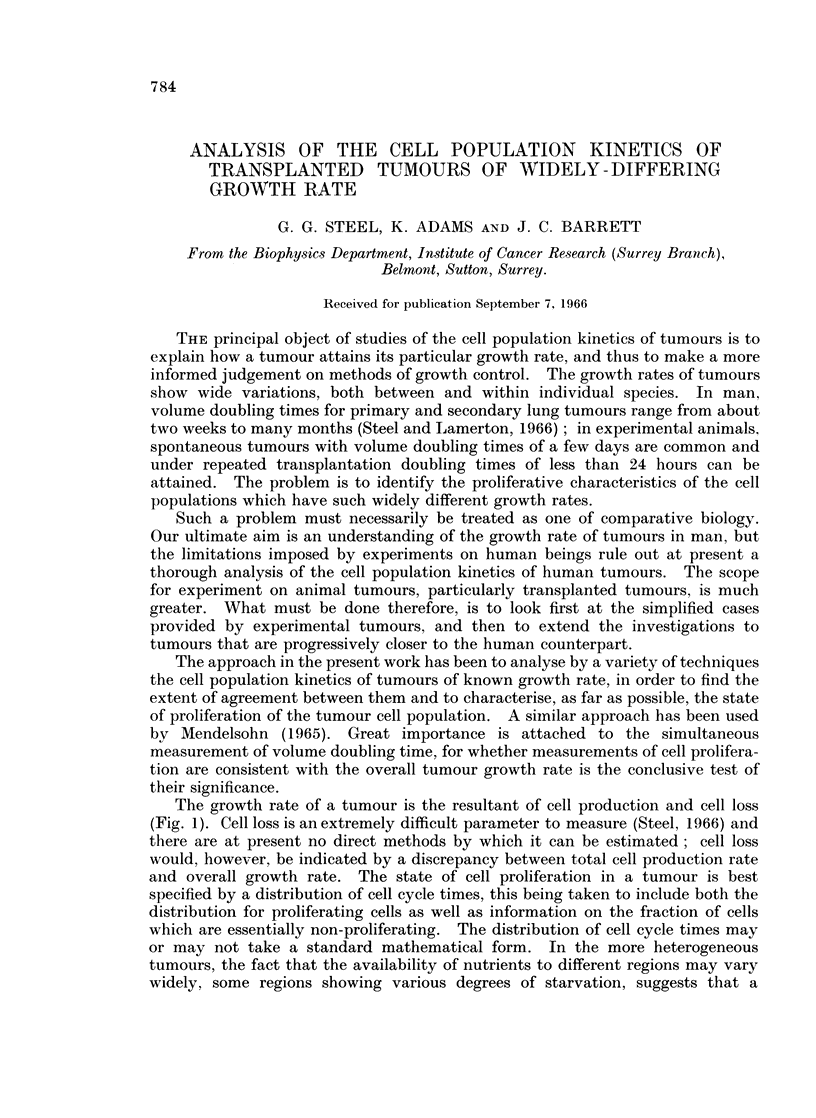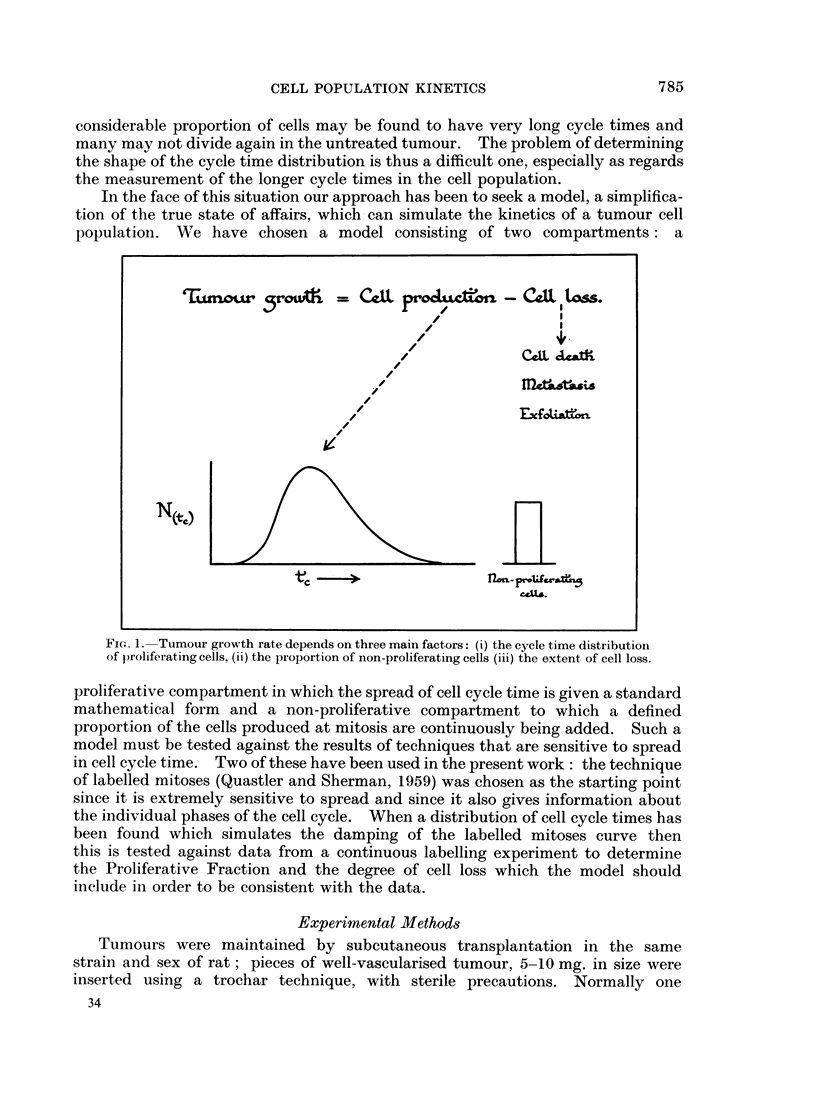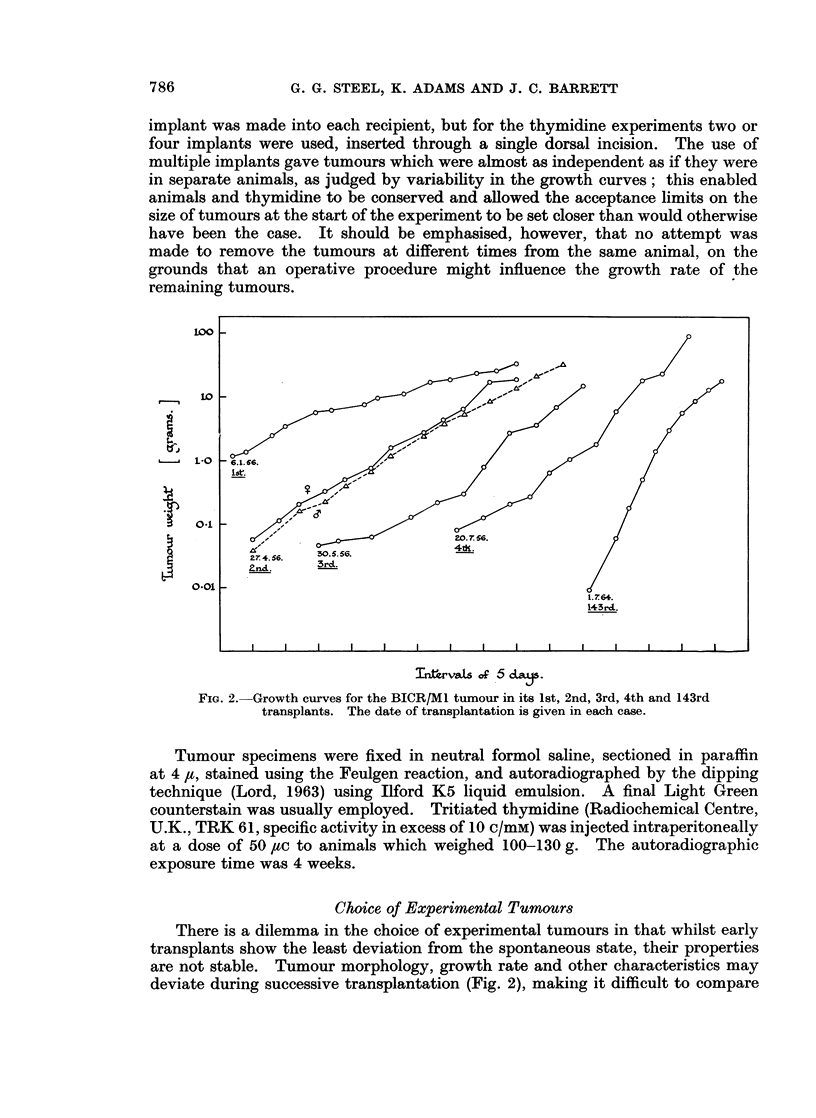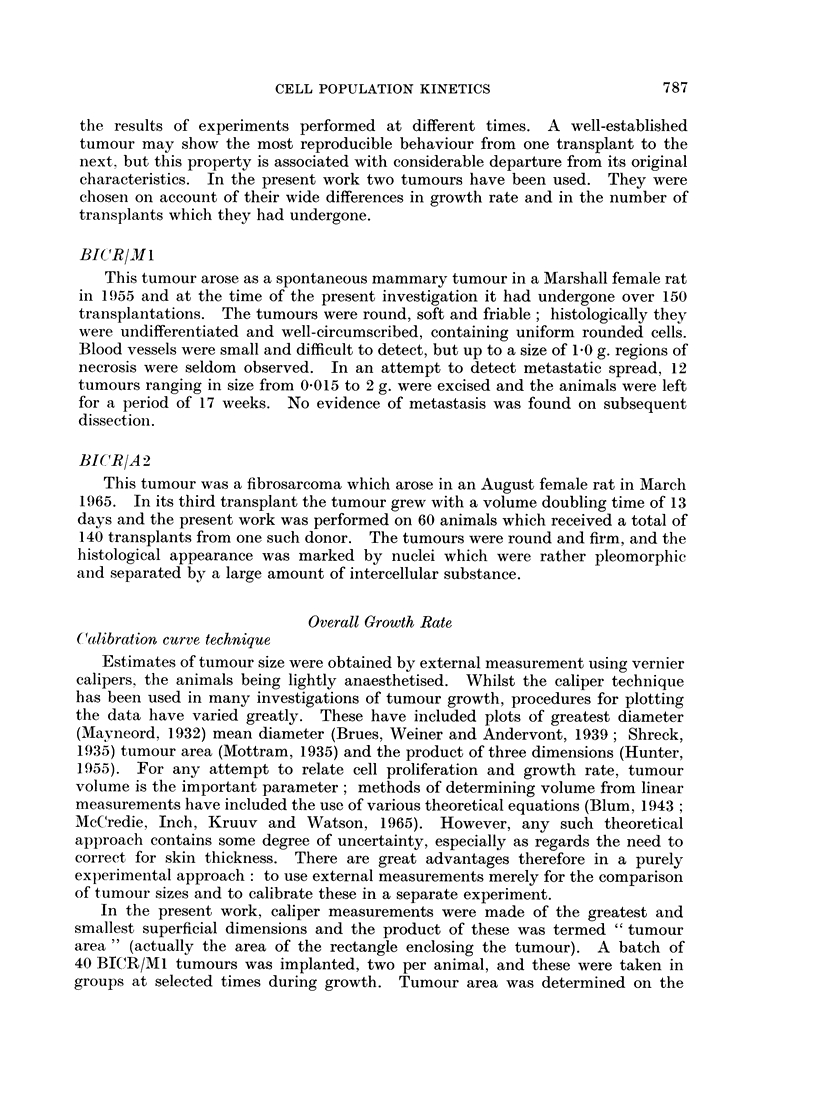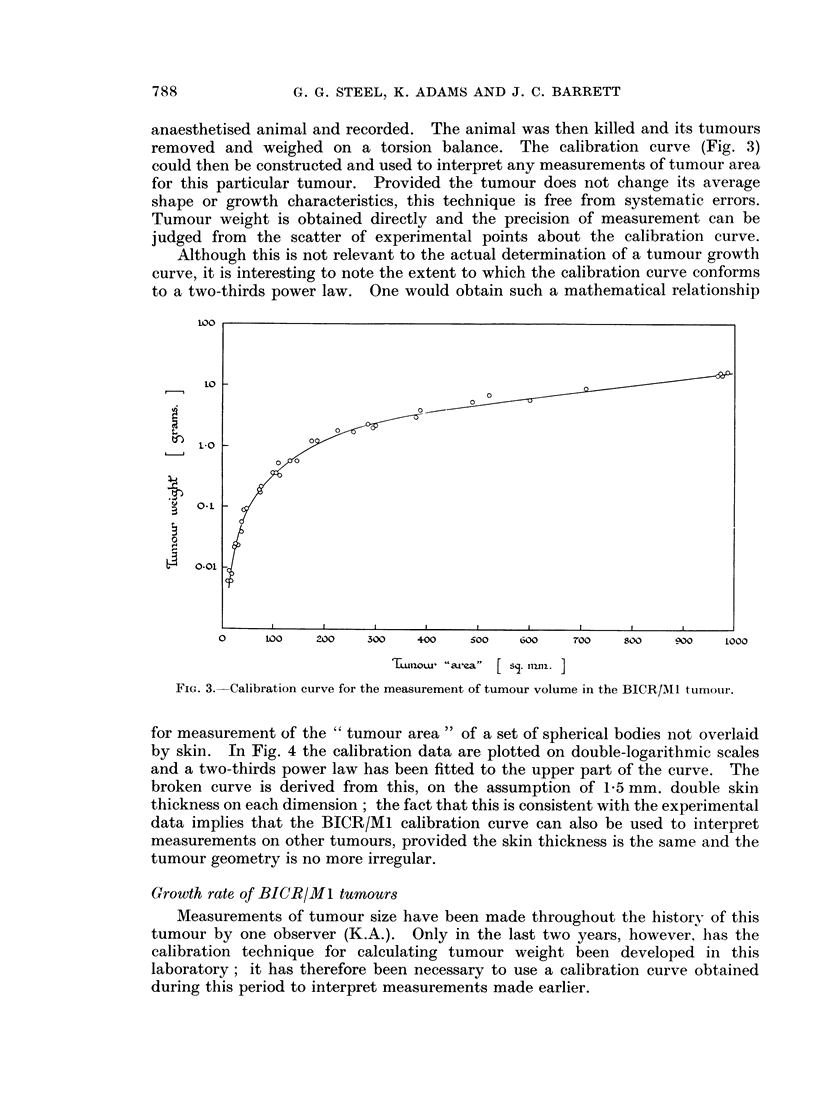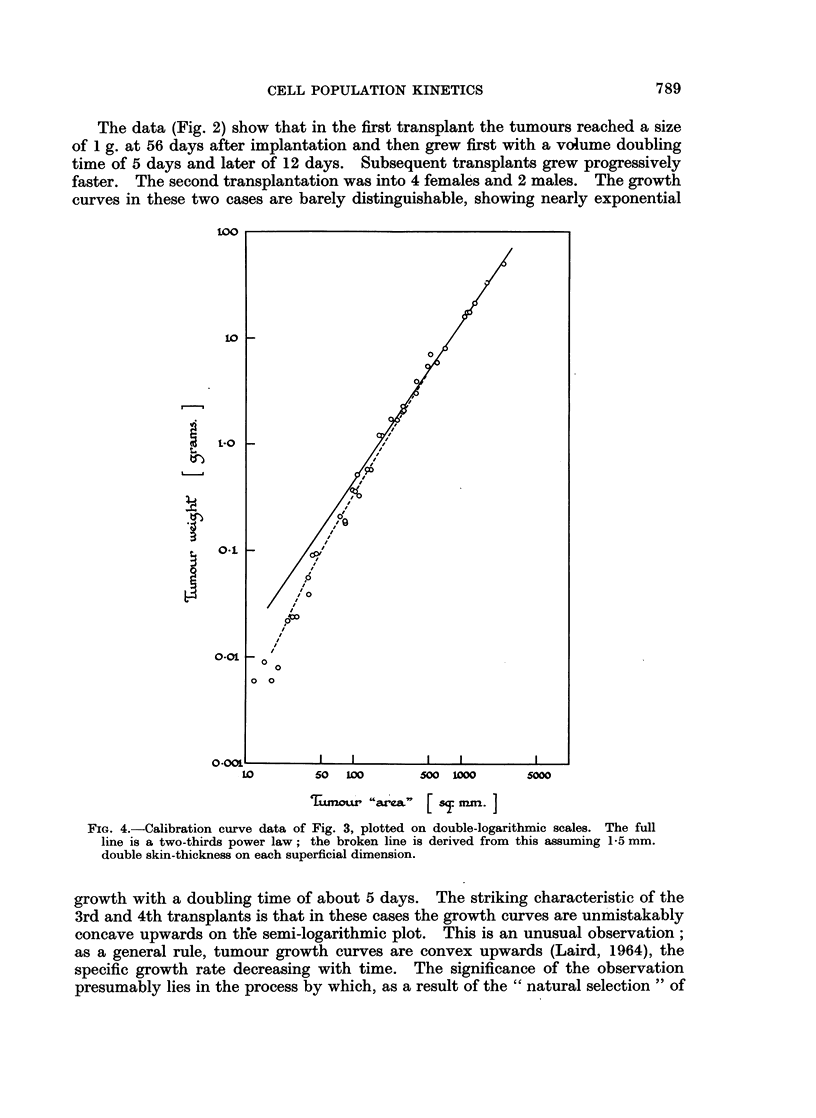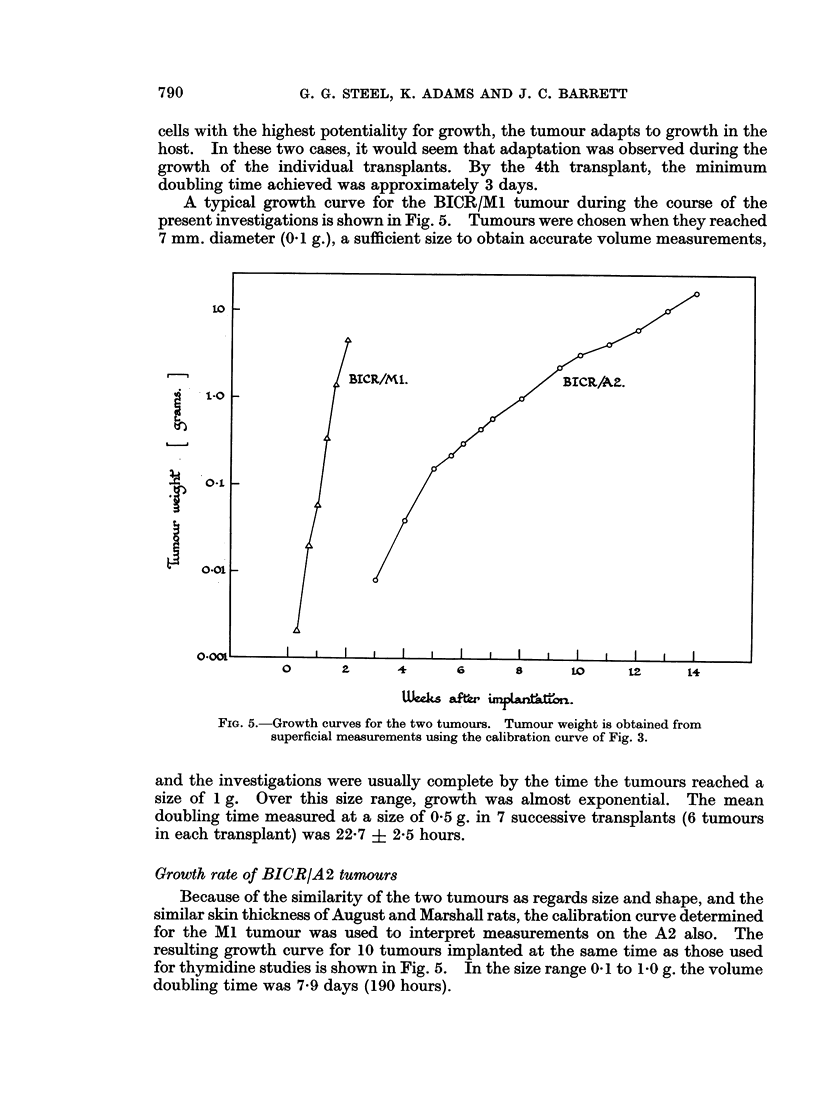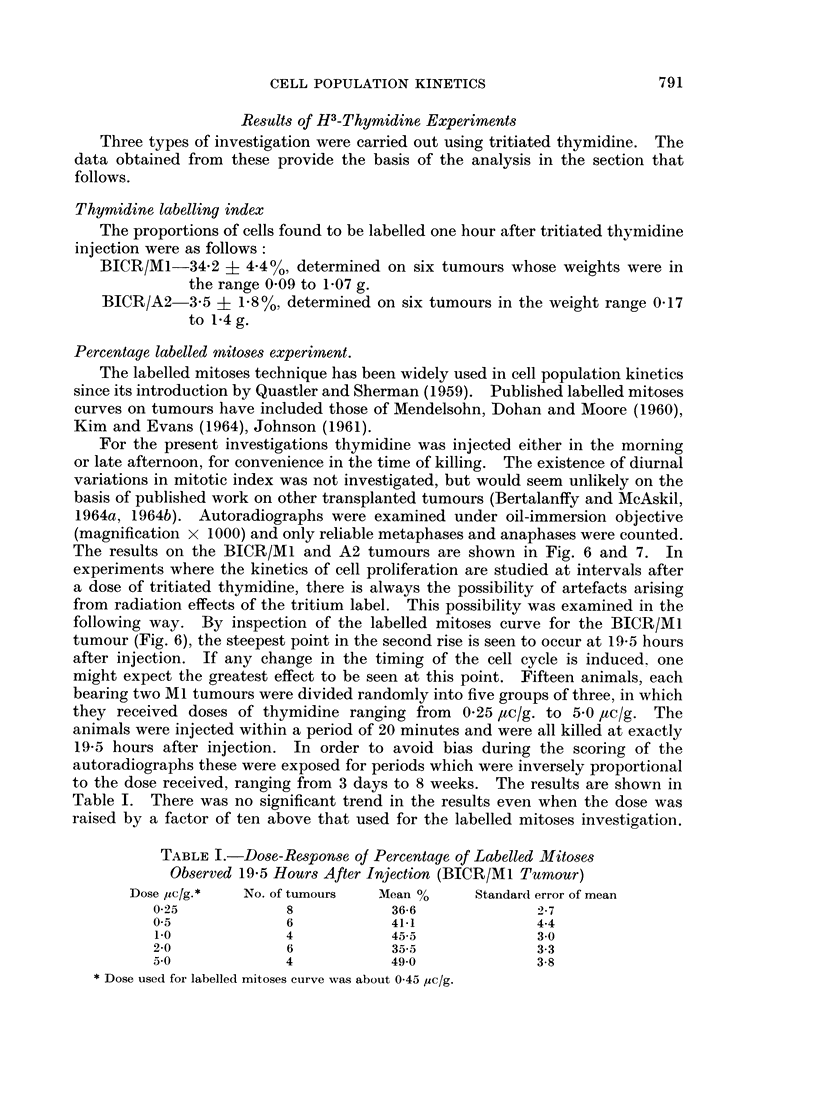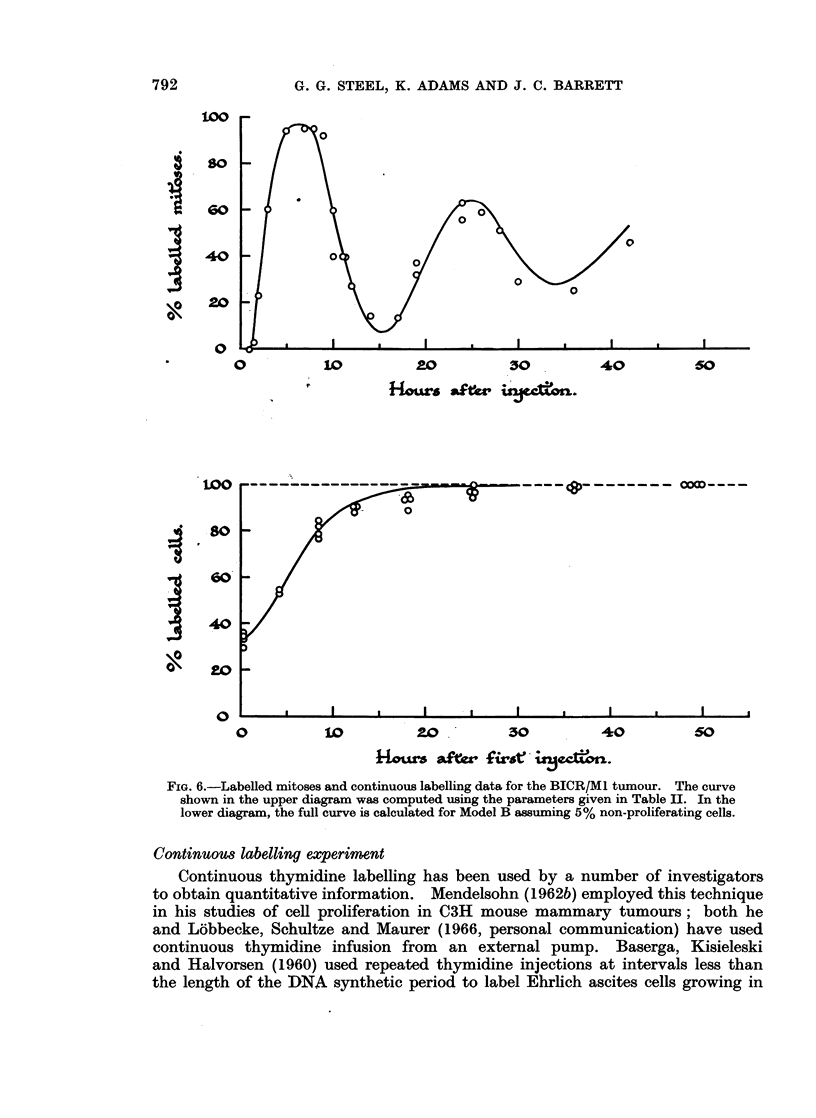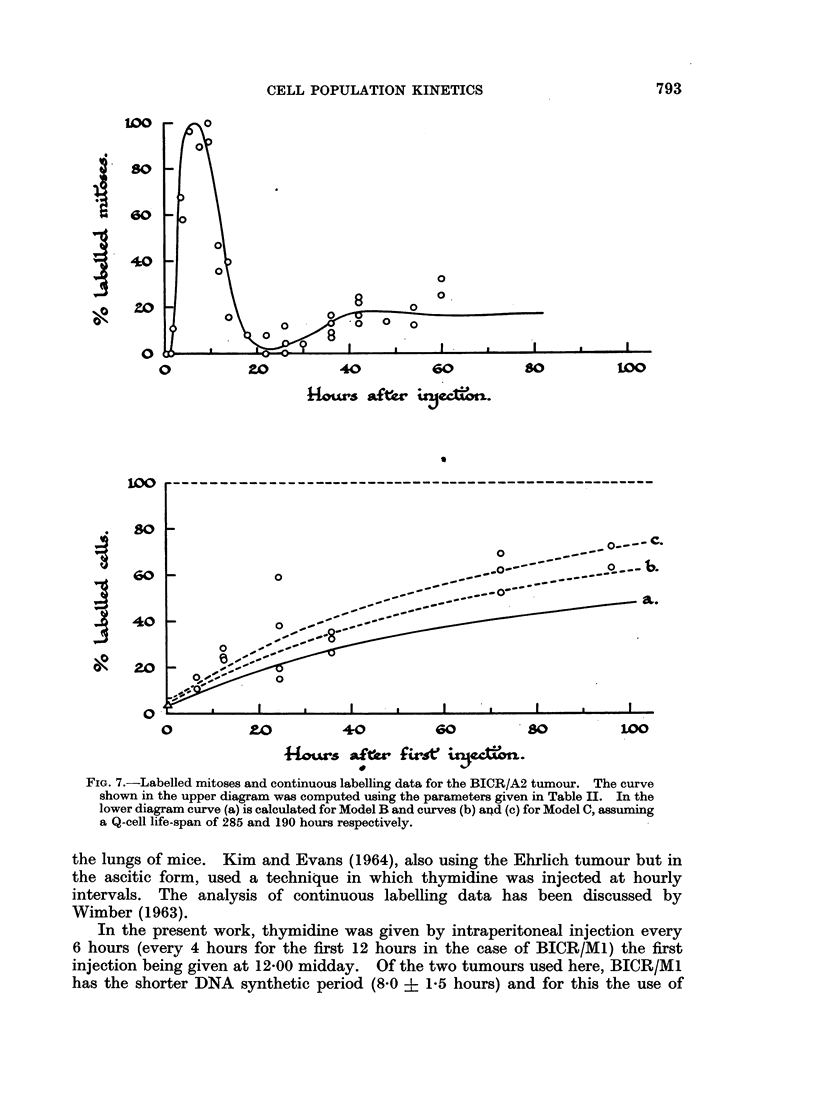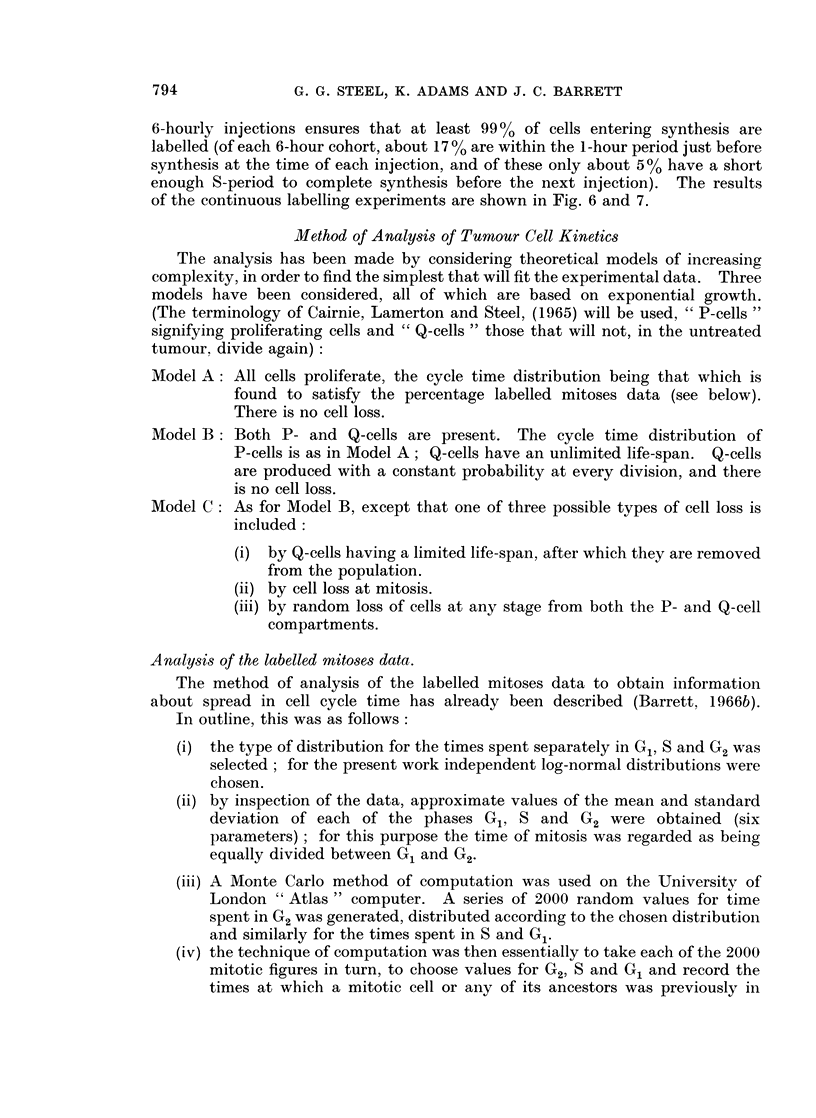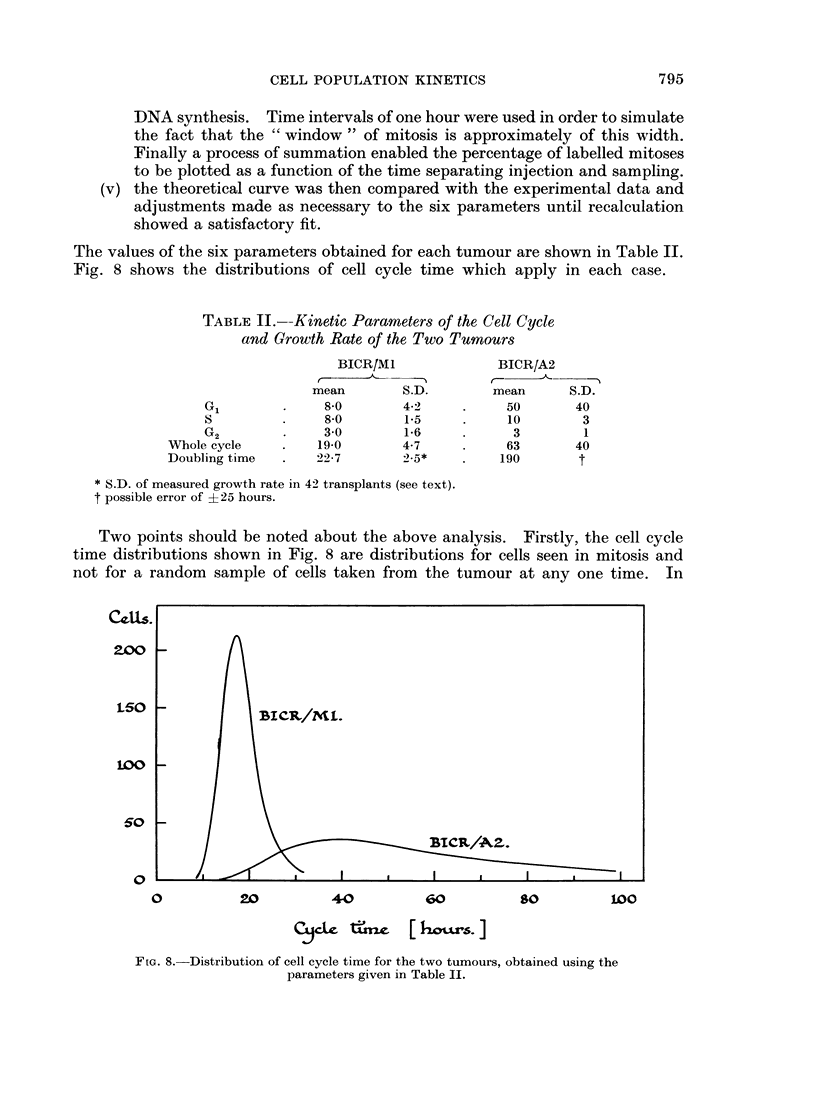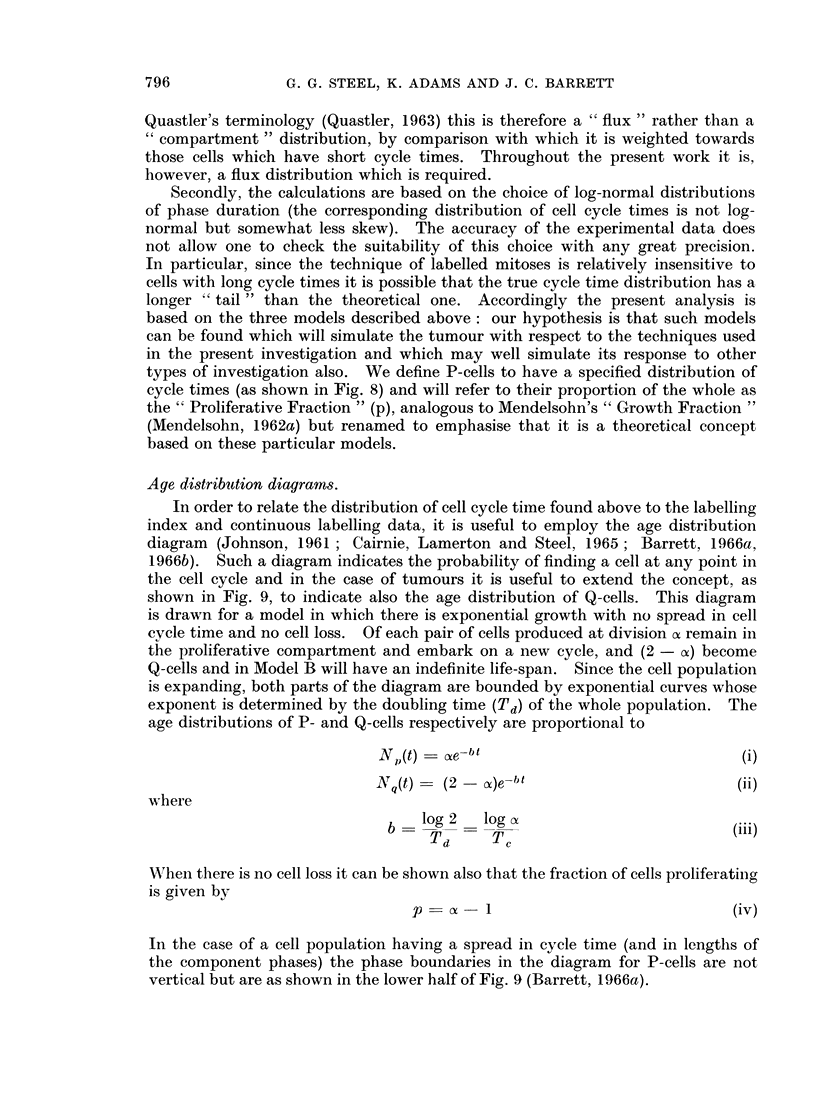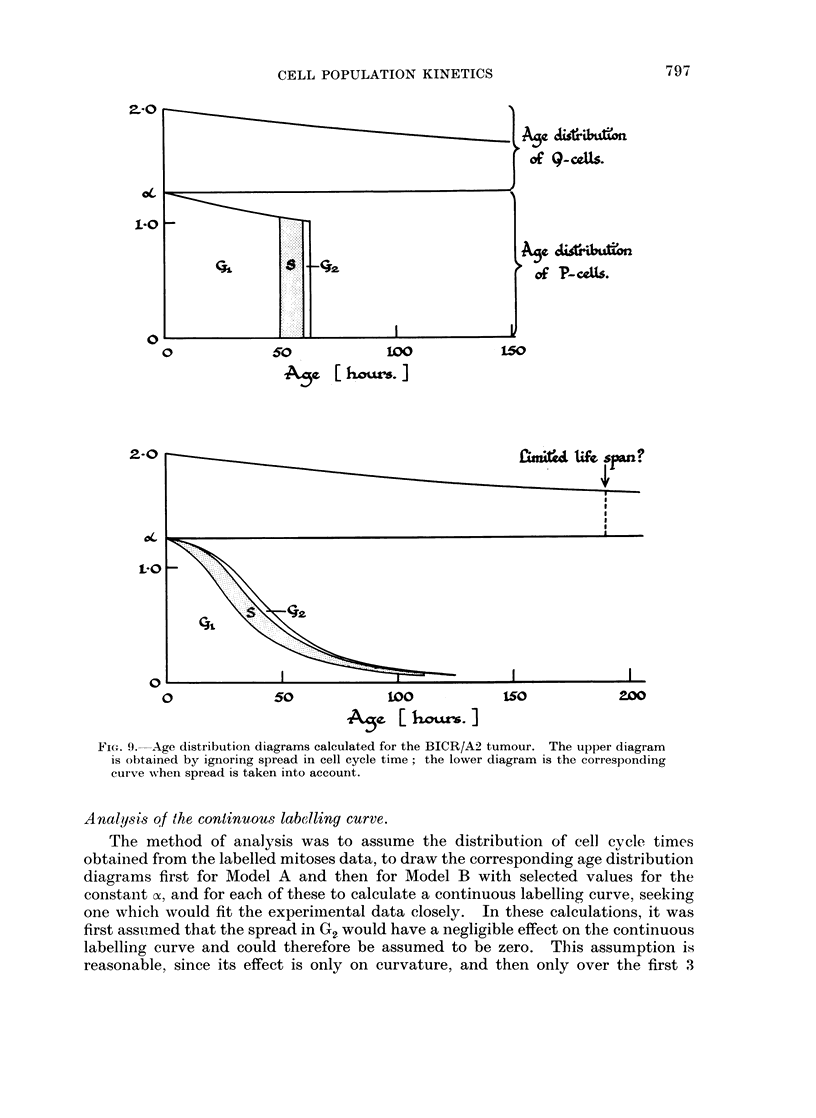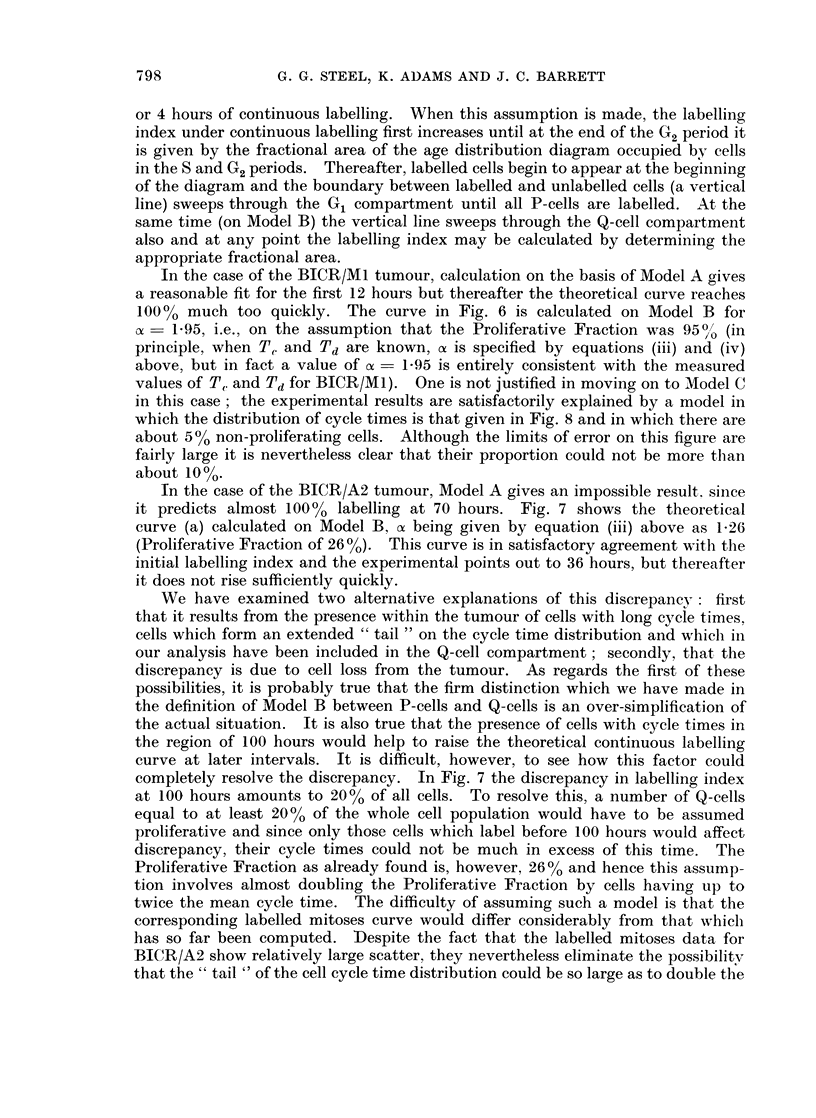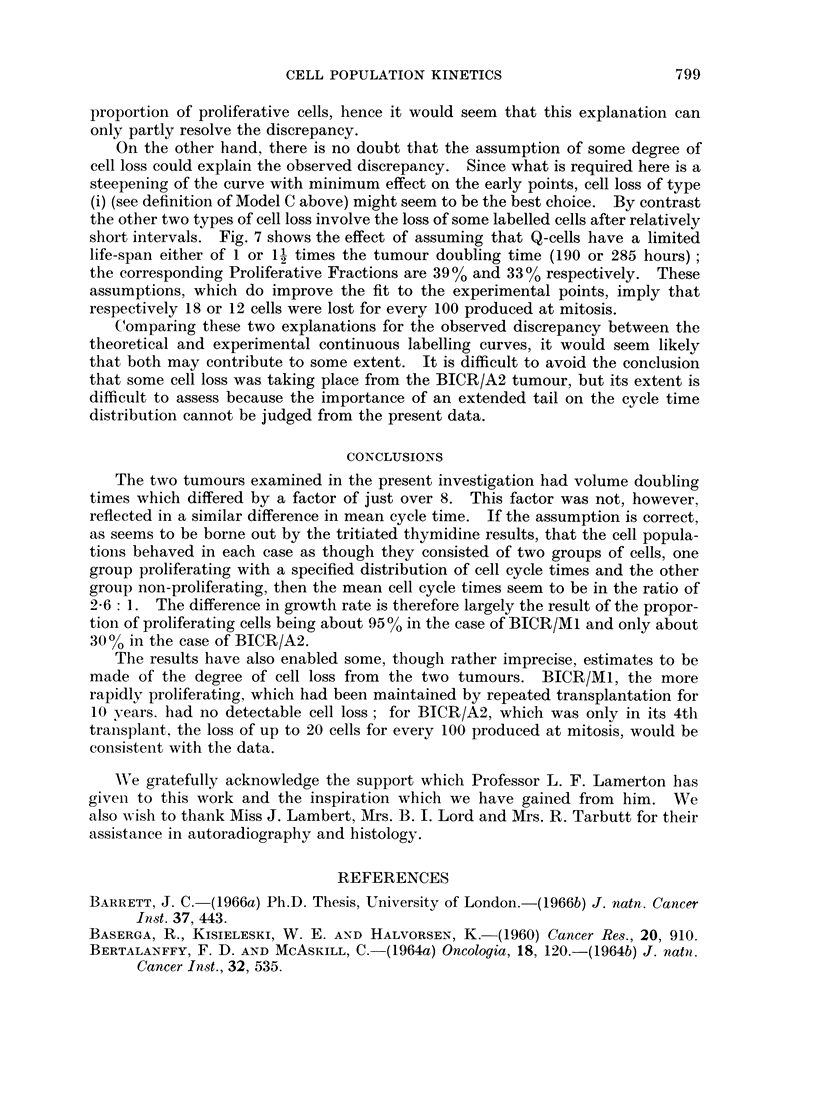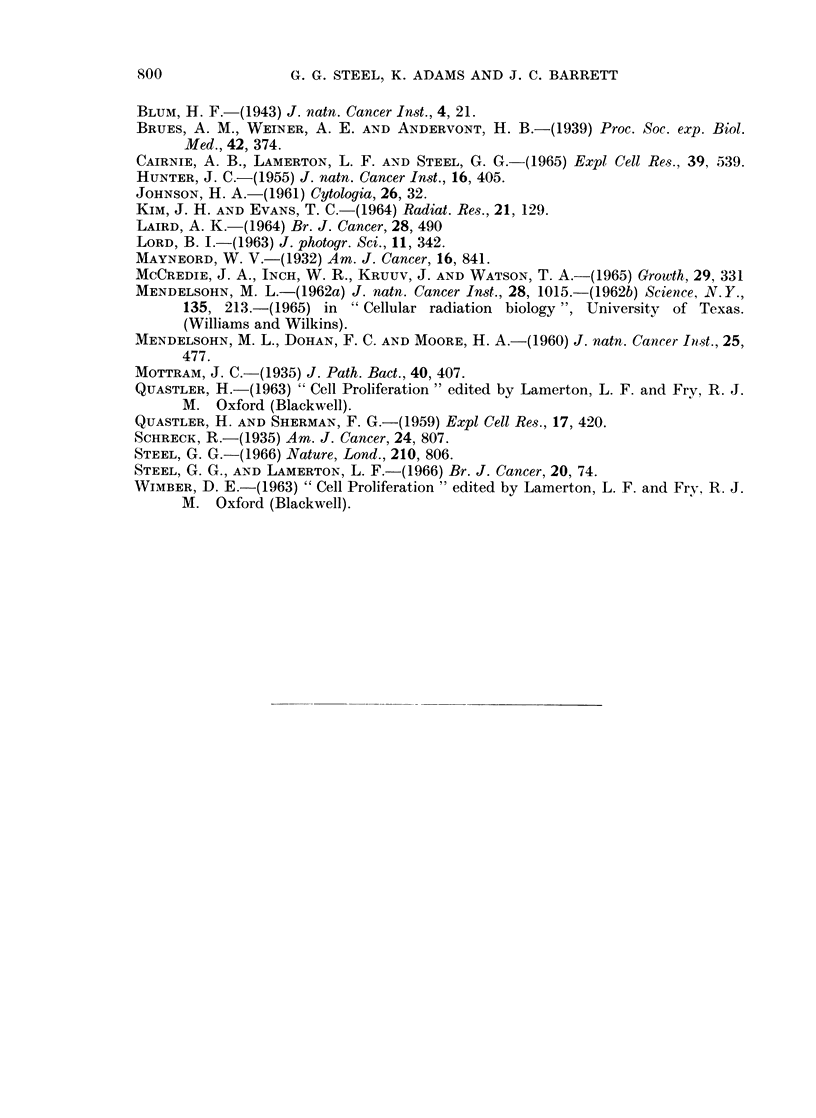# Analysis of the cell population kinetics of transplanted tumours of widely-differing growth rate.

**DOI:** 10.1038/bjc.1966.90

**Published:** 1966-12

**Authors:** G. G. Steel, K. Adams, J. C. Barrett


### ANALYSIS OF THE CELL POPULATION KINETICS OF TRANSPLANTED TUMOURS OF WIDELY-DIFFERING GROWTH RATE

G. G. STEEL, K. ADAMS AND J. C. BARRETT

*From the Biophysics Department, Institute of Cancer Research (Surrey Branch), Belmont, Sutton, Surrey.*

Received for publication September 7, 1966

THE principal object of studies of the cell population kinetics of tumours is to explain how a tumour attains its particular growth rate, and thus to make a more informed judgement on methods of growth control. The growth rates of tumours show wide variations, both between and within individual species. In man, volume doubling times for primary and secondary lung tumours range from about two weeks to many months (Steel and Lamerton, 1966) ; in experimental animals, spontaneous tumours with volume doubling times of a few days are common and under repeated transplantation doubling times of less than 24 hours can be attained. The problem is to identify the proliferative characteristics of the cell populations which have such widely different growth rates.

Such a problem must necessarily be treated as one of comparative biology. Our ultimate aim is an understanding of the growth rate of tumours in man, but the limitations imposed by experiments on human beings rule out at present a thorough analysis of the cell population kinetics of human tumours. The scope for experiment on animal tumours, particularly transplanted tumours, is much greater. What must be done therefore, is to look first at the simplified cases provided by experimental tumours, and then to extend the investigations to tumours that are progressively closer to the human counterpart.

The approach in the present work has been to analyse by a variety of techniques the cell population kinetics of tumours of known growth rate, in order to find the extent of agreement between them and to characterise, as far as possible, the state of proliferation of the tumour cell population. A similar approach has been used by Mendelsohn (1965). Great importance is attached to the simultaneous measurement of volume doubling time, for whether measurements of cell proliferation are consistent with the overall tumour growth rate is the conclusive test of their significance.

The growth rate of a tumour is the resultant of cell production and cell loss (Fig. 1). Cell loss is an extremely difficult parameter to measure (Steel, 1966) and there are at present no direct methods by which it can be estimated ; cell loss would, however, be indicated by a discrepancy between total cell production rate and overall growth rate. The state of cell proliferation in a tumour is best specified by a distribution of cell cycle times, this being taken to include both the distribution for proliferating cells as well as information on the fraction of cells which are essentially non-proliferating. The distribution of cell cycle times may or may not take a standard mathematical form. In the more heterogeneous tumours, the fact that the availability of nutrients to different regions may vary widely, some regions showing various degrees of starvation, suggests that a considerable proportion of cells may be found to have very long cycle times and many may not divide again in the untreated tumour. The problem of determining the shape of the cycle time distribution is thus a difficult one, especially as regards the measurement of the longer cycle times in the cell population.

In the face of this situation our approach has been to seek a model, a simplification of the true state of affairs, which can simulate the kinetics of a tumour cell population. We have chosen a model consisting of two compartments: a

FIG. 1.—Tumour growth rate depends on three main factors: (i) the cycle time distribution of proliferating cells, (ii) the proportion of non-proliferating cells (iii) the extent of cell loss.

proliferative compartment in which the spread of cell cycle time is given a standard mathematical form and a non-proliferative compartment to which a defined proportion of the cells produced at mitosis are continuously being added. Such a model must be tested against the results of techniques that are sensitive to spread in cell cycle time. Two of these have been used in the present work: the technique of labelled mitoses (Quastler and Sherman, 1959) was chosen as the starting point since it is extremely sensitive to spread and since it also gives information about the individual phases of the cell cycle. When a distribution of cell cycle times has been found which simulates the damping of the labelled mitoses curve then this is tested against data from a continuous labelling experiment to determine the Proliferative Fraction and the degree of cell loss which the model should include in order to be consistent with the data.

### *Experimental Methods*

Tumours were maintained by subcutaneous transplantation in the same strain and sex of rat; pieces of well-vascularised tumour, 5–10 mg. in size were inserted using a trochar technique, with sterile precautions. Normally one implant was made into each recipient, but for the thymidine experiments two or four implants were used, inserted through a single dorsal incision. The use of multiple implants gave tumours which were almost as independent as if they were in separate animals, as judged by variability in the growth curves ; this enabled animals and thymidine to be conserved and allowed the acceptance limits on the size of tumours at the start of the experiment to be set closer than would otherwise have been the case. It should be emphasised, however, that no attempt was made to remove the tumours at different times from the same animal, on the grounds that an operative procedure might influence the growth rate of the remaining tumours.

FIG. 2.—Growth curves for the BICR/M1 tumour in its 1st, 2nd, 3rd, 4th and 143rd transplants. The date of transplantation is given in each case.

Tumour specimens were fixed in neutral formol saline, sectioned in paraffin at 4 $\mu$, stained using the Feulgen reaction, and autoradiographed by the dipping technique (Lord, 1963) using Ilford K5 liquid emulsion. A final Light Green counterstain was usually employed. Tritiated thymidine (Radiochemical Centre, U.K., TRK 61, specific activity in excess of 10 c/mM) was injected intraperitoneally at a dose of 50 $\mu$c to animals which weighed 100–130 g. The autoradiographic exposure time was 4 weeks.

### *Choice of Experimental Tumours*

There is a dilemma in the choice of experimental tumours in that whilst early transplants show the least deviation from the spontaneous state, their properties are not stable. Tumour morphology, growth rate and other characteristics may deviate during successive transplantation (Fig. 2), making it difficult to compare the results of experiments performed at different times. A well-established tumour may show the most reproducible behaviour from one transplant to the next, but this property is associated with considerable departure from its original characteristics. In the present work two tumours have been used. They were chosen on account of their wide differences in growth rate and in the number of transplants which they had undergone.

*BICR/M1*

This tumour arose as a spontaneous mammary tumour in a Marshall female rat in 1955 and at the time of the present investigation it had undergone over 150 transplantations. The tumours were round, soft and friable ; histologically they were undifferentiated and well-circumscribed, containing uniform rounded cells. Blood vessels were small and difficult to detect, but up to a size of 1·0 g. regions of necrosis were seldom observed. In an attempt to detect metastatic spread, 12 tumours ranging in size from 0·015 to 2 g. were excised and the animals were left for a period of 17 weeks. No evidence of metastasis was found on subsequent dissection.

*BICR/A2*

This tumour was a fibrosarcoma which arose in an August female rat in March 1965. In its third transplant the tumour grew with a volume doubling time of 13 days and the present work was performed on 60 animals which received a total of 140 transplants from one such donor. The tumours were round and firm, and the histological appearance was marked by nuclei which were rather pleomorphic and separated by a large amount of intercellular substance.

### *Overall Growth Rate*

*Calibration curve technique*

Estimates of tumour size were obtained by external measurement using vernier calipers, the animals being lightly anaesthetised. Whilst the caliper technique has been used in many investigations of tumour growth, procedures for plotting the data have varied greatly. These have included plots of greatest diameter (Mayneord, 1932) mean diameter (Brues, Weiner and Andervont, 1939 ; Shreck, 1935) tumour area (Mottram, 1935) and the product of three dimensions (Hunter, 1955). For any attempt to relate cell proliferation and growth rate, tumour volume is the important parameter ; methods of determining volume from linear measurements have included the use of various theoretical equations (Blum, 1943 ; McCredie, Inch, Kruuv and Watson, 1965). However, any such theoretical approach contains some degree of uncertainty, especially as regards the need to correct for skin thickness. There are great advantages therefore in a purely experimental approach : to use external measurements merely for the comparison of tumour sizes and to calibrate these in a separate experiment.

In the present work, caliper measurements were made of the greatest and smallest superficial dimensions and the product of these was termed " tumour area " (actually the area of the rectangle enclosing the tumour). A batch of 40 BICR/M1 tumours was implanted, two per animal, and these were taken in groups at selected times during growth. Tumour area was determined on the anaesthetised animal and recorded. The animal was then killed and its tumours removed and weighed on a torsion balance. The calibration curve (Fig. 3) could then be constructed and used to interpret any measurements of tumour area for this particular tumour. Provided the tumour does not change its average shape or growth characteristics, this technique is free from systematic errors. Tumour weight is obtained directly and the precision of measurement can be judged from the scatter of experimental points about the calibration curve.

Although this is not relevant to the actual determination of a tumour growth curve, it is interesting to note the extent to which the calibration curve conforms to a two-thirds power law. One would obtain such a mathematical relationship

Fig. 3.—Calibration curve for the measurement of tumour volume in the BICR/M1 tumour.

for measurement of the " tumour area " of a set of spherical bodies not overlaid by skin. In Fig. 4 the calibration data are plotted on double-logarithmic scales and a two-thirds power law has been fitted to the upper part of the curve. The broken curve is derived from this, on the assumption of 1·5 mm. double skin thickness on each dimension ; the fact that this is consistent with the experimental data implies that the BICR/M1 calibration curve can also be used to interpret measurements on other tumours, provided the skin thickness is the same and the tumour geometry is no more irregular.

*Growth rate of BICR/M*1 *tumours*

Measurements of tumour size have been made throughout the history of this tumour by one observer (K.A.). Only in the last two years, however, has the calibration technique for calculating tumour weight been developed in this laboratory ; it has therefore been necessary to use a calibration curve obtained during this period to interpret measurements made earlier.

The data (Fig. 2) show that in the first transplant the tumours reached a size of 1 g. at 56 days after implantation and then grew first with a volume doubling time of 5 days and later of 12 days. Subsequent transplants grew progressively faster. The second transplantation was into 4 females and 2 males. The growth curves in these two cases are barely distinguishable, showing nearly exponential

Fig. 4.—Calibration curve data of Fig. 3, plotted on double-logarithmic scales. The full line is a two-thirds power law; the broken line is derived from this assuming 1·5 mm. double skin-thickness on each superficial dimension.

growth with a doubling time of about 5 days. The striking characteristic of the 3rd and 4th transplants is that in these cases the growth curves are unmistakably concave upwards on the semi-logarithmic plot. This is an unusual observation; as a general rule, tumour growth curves are convex upwards (Laird, 1964), the specific growth rate decreasing with time. The significance of the observation presumably lies in the process by which, as a result of the " natural selection " of cells with the highest potentiality for growth, the tumour adapts to growth in the host. In these two cases, it would seem that adaptation was observed during the growth of the individual transplants. By the 4th transplant, the minimum doubling time achieved was approximately 3 days.

A typical growth curve for the BICR/M1 tumour during the course of the present investigations is shown in Fig. 5. Tumours were chosen when they reached 7 mm. diameter (0·1 g.), a sufficient size to obtain accurate volume measurements,

FIG. 5.—Growth curves for the two tumours. Tumour weight is obtained from superficial measurements using the calibration curve of Fig. 3.

and the investigations were usually complete by the time the tumours reached a size of 1 g. Over this size range, growth was almost exponential. The mean doubling time measured at a size of 0·5 g. in 7 successive transplants (6 tumours in each transplant) was 22·7 $\pm$ 2·5 hours.

*Growth rate of BICR/A2 tumours*

Because of the similarity of the two tumours as regards size and shape, and the similar skin thickness of August and Marshall rats, the calibration curve determined for the M1 tumour was used to interpret measurements on the A2 also. The resulting growth curve for 10 tumours implanted at the same time as those used for thymidine studies is shown in Fig. 5. In the size range 0·1 to 1·0 g. the volume doubling time was 7·9 days (190 hours).

### *Results of $H^3$-Thymidine Experiments*

Three types of investigation were carried out using tritiated thymidine. The data obtained from these provide the basis of the analysis in the section that follows.

*Thymidine labelling index*

The proportions of cells found to be labelled one hour after tritiated thymidine injection were as follows :

BICR/M1—34·2 ± 4·4%, determined on six tumours whose weights were in the range 0·09 to 1·07 g.

BICR/A2—3·5 ± 1·8%, determined on six tumours in the weight range 0·17 to 1·4 g.

*Percentage labelled mitoses experiment.*

The labelled mitoses technique has been widely used in cell population kinetics since its introduction by Quastler and Sherman (1959). Published labelled mitoses curves on tumours have included those of Mendelsohn, Dohan and Moore (1960), Kim and Evans (1964), Johnson (1961).

For the present investigations thymidine was injected either in the morning or late afternoon, for convenience in the time of killing. The existence of diurnal variations in mitotic index was not investigated, but would seem unlikely on the basis of published work on other transplanted tumours (Bertalanffy and McAskil, 1964*a*, 1964*b*). Autoradiographs were examined under oil-immersion objective (magnification × 1000) and only reliable metaphases and anaphases were counted. The results on the BICR/M1 and A2 tumours are shown in Fig. 6 and 7. In experiments where the kinetics of cell proliferation are studied at intervals after a dose of tritiated thymidine, there is always the possibility of artefacts arising from radiation effects of the tritium label. This possibility was examined in the following way. By inspection of the labelled mitoses curve for the BICR/M1 tumour (Fig. 6), the steepest point in the second rise is seen to occur at 19·5 hours after injection. If any change in the timing of the cell cycle is induced, one might expect the greatest effect to be seen at this point. Fifteen animals, each bearing two M1 tumours were divided randomly into five groups of three, in which they received doses of thymidine ranging from 0·25 μc/g. to 5·0 μc/g. The animals were injected within a period of 20 minutes and were all killed at exactly 19·5 hours after injection. In order to avoid bias during the scoring of the autoradiographs these were exposed for periods which were inversely proportional to the dose received, ranging from 3 days to 8 weeks. The results are shown in Table I. There was no significant trend in the results even when the dose was raised by a factor of ten above that used for the labelled mitoses investigation.

TABLE I.—*Dose-Response of Percentage of Labelled Mitoses Observed* 19·5 *Hours After Injection* (BICR/M1 *Tumour*)

| Dose μc/g.* | No. of tumours | Mean % | Standard error of mean |
|---|---|---|---|
| 0·25 | 8 | 36·6 | 2·7 |
| 0·5 | 6 | 41·1 | 4·4 |
| 1·0 | 4 | 45·5 | 3·0 |
| 2·0 | 6 | 35·5 | 3·3 |
| 5·0 | 4 | 49·0 | 3·8 |

* Dose used for labelled mitoses curve was about 0·45 μc/g.

FIG. 6.—Labelled mitoses and continuous labelling data for the BICR/M1 tumour. The curve shown in the upper diagram was computed using the parameters given in Table II. In the lower diagram, the full curve is calculated for Model B assuming 5% non-proliferating cells.

*Continuous labelling experiment*

Continuous thymidine labelling has been used by a number of investigators to obtain quantitative information. Mendelsohn (1962*b*) employed this technique in his studies of cell proliferation in C3H mouse mammary tumours ; both he and Löbbecke, Schultze and Maurer (1966, personal communication) have used continuous thymidine infusion from an external pump. Baserga, Kisieleski and Halvorsen (1960) used repeated thymidine injections at intervals less than the length of the DNA synthetic period to label Ehrlich ascites cells growing in

FIG. 7.—Labelled mitoses and continuous labelling data for the BICR/A2 tumour. The curve shown in the upper diagram was computed using the parameters given in Table II. In the lower diagram curve (a) is calculated for Model B and curves (b) and (c) for Model C, assuming a Q-cell life-span of 285 and 190 hours respectively.

the lungs of mice. Kim and Evans (1964), also using the Ehrlich tumour but in the ascitic form, used a technique in which thymidine was injected at hourly intervals. The analysis of continuous labelling data has been discussed by Wimber (1963).

In the present work, thymidine was given by intraperitoneal injection every 6 hours (every 4 hours for the first 12 hours in the case of BICR/M1) the first injection being given at 12·00 midday. Of the two tumours used here, BICR/M1 has the shorter DNA synthetic period (8·0 $\pm$ 1·5 hours) and for this the use of

6-hourly injections ensures that at least 99% of cells entering synthesis are labelled (of each 6-hour cohort, about 17% are within the 1-hour period just before synthesis at the time of each injection, and of these only about 5% have a short enough S-period to complete synthesis before the next injection). The results of the continuous labelling experiments are shown in Fig. 6 and 7.

*Method of Analysis of Tumour Cell Kinetics*

The analysis has been made by considering theoretical models of increasing complexity, in order to find the simplest that will fit the experimental data. Three models have been considered, all of which are based on exponential growth. (The terminology of Cairnie, Lamerton and Steel, (1965) will be used, " P-cells " signifying proliferating cells and " Q-cells " those that will not, in the untreated tumour, divide again) :

Model A : All cells proliferate, the cycle time distribution being that which is found to satisfy the percentage labelled mitoses data (see below). There is no cell loss.

Model B : Both P- and Q-cells are present. The cycle time distribution of P-cells is as in Model A ; Q-cells have an unlimited life-span. Q-cells are produced with a constant probability at every division, and there is no cell loss.

Model C : As for Model B, except that one of three possible types of cell loss is included :

- (i) by Q-cells having a limited life-span, after which they are removed from the population.
- (ii) by cell loss at mitosis.
- (iii) by random loss of cells at any stage from both the P- and Q-cell compartments.

*Analysis of the labelled mitoses data.*

The method of analysis of the labelled mitoses data to obtain information about spread in cell cycle time has already been described (Barrett, 1966*b*). In outline, this was as follows :

- (i) the type of distribution for the times spent separately in $G_1$, S and $G_2$ was selected ; for the present work independent log-normal distributions were chosen.
- (ii) by inspection of the data, approximate values of the mean and standard deviation of each of the phases $G_1$, S and $G_2$ were obtained (six parameters) ; for this purpose the time of mitosis was regarded as being equally divided between $G_1$ and $G_2$.
- (iii) A Monte Carlo method of computation was used on the University of London " Atlas " computer. A series of 2000 random values for time spent in $G_2$ was generated, distributed according to the chosen distribution and similarly for the times spent in S and $G_1$.
- (iv) the technique of computation was then essentially to take each of the 2000 mitotic figures in turn, to choose values for $G_2$, S and $G_1$ and record the times at which a mitotic cell or any of its ancestors was previously in

DNA synthesis. Time intervals of one hour were used in order to simulate the fact that the " window " of mitosis is approximately of this width. Finally a process of summation enabled the percentage of labelled mitoses to be plotted as a function of the time separating injection and sampling.

(v) the theoretical curve was then compared with the experimental data and adjustments made as necessary to the six parameters until recalculation showed a satisfactory fit.

The values of the six parameters obtained for each tumour are shown in Table II. Fig. 8 shows the distributions of cell cycle time which apply in each case.

TABLE II.—*Kinetic Parameters of the Cell Cycle and Growth Rate of the Two Tumours*

| | BICR/M1 | | BICR/A2 | |
|---|---|---|---|---|
| | mean | S.D. | mean | S.D. |
| $G_1$ | 8·0 | 4·2 | 50 | 40 |
| S | 8·0 | 1·5 | 10 | 3 |
| $G_2$ | 3·0 | 1·6 | 3 | 1 |
| Whole cycle | 19·0 | 4·7 | 63 | 40 |
| Doubling time | 22·7 | 2·5* | 190 | † |

* S.D. of measured growth rate in 42 transplants (see text).
† possible error of $\pm 25$ hours.

Two points should be noted about the above analysis. Firstly, the cell cycle time distributions shown in Fig. 8 are distributions for cells seen in mitosis and not for a random sample of cells taken from the tumour at any one time. In

FIG. 8.—Distribution of cell cycle time for the two tumours, obtained using the parameters given in Table II.

Quastler's terminology (Quastler, 1963) this is therefore a " flux " rather than a " compartment " distribution, by comparison with which it is weighted towards those cells which have short cycle times. Throughout the present work it is, however, a flux distribution which is required.

Secondly, the calculations are based on the choice of log-normal distributions of phase duration (the corresponding distribution of cell cycle times is not log-normal but somewhat less skew). The accuracy of the experimental data does not allow one to check the suitability of this choice with any great precision. In particular, since the technique of labelled mitoses is relatively insensitive to cells with long cycle times it is possible that the true cycle time distribution has a longer " tail " than the theoretical one. Accordingly the present analysis is based on the three models described above : our hypothesis is that such models can be found which will simulate the tumour with respect to the techniques used in the present investigation and which may well simulate its response to other types of investigation also. We define P-cells to have a specified distribution of cycle times (as shown in Fig. 8) and will refer to their proportion of the whole as the " Proliferative Fraction " (p), analogous to Mendelsohn's " Growth Fraction " (Mendelsohn, 1962*a*) but renamed to emphasise that it is a theoretical concept based on these particular models.

*Age distribution diagrams.*

In order to relate the distribution of cell cycle time found above to the labelling index and continuous labelling data, it is useful to employ the age distribution diagram (Johnson, 1961 ; Cairnie, Lamerton and Steel, 1965 ; Barrett, 1966*a*, 1966*b*). Such a diagram indicates the probability of finding a cell at any point in the cell cycle and in the case of tumours it is useful to extend the concept, as shown in Fig. 9, to indicate also the age distribution of Q-cells. This diagram is drawn for a model in which there is exponential growth with no spread in cell cycle time and no cell loss. Of each pair of cells produced at division $\alpha$ remain in the proliferative compartment and embark on a new cycle, and $(2 - \alpha)$ become Q-cells and in Model B will have an indefinite life-span. Since the cell population is expanding, both parts of the diagram are bounded by exponential curves whose exponent is determined by the doubling time ($T_d$) of the whole population. The age distributions of P- and Q-cells respectively are proportional to

$$N_p(t) = \alpha e^{-bt} \qquad \text{(i)}$$

$$N_q(t) = (2 - \alpha)e^{-bt} \qquad \text{(ii)}$$

where

$$b = \frac{\log 2}{T_d} = \frac{\log \alpha}{T_c} \qquad \text{(iii)}$$

When there is no cell loss it can be shown also that the fraction of cells proliferating is given by

$$p = \alpha - 1 \qquad \text{(iv)}$$

In the case of a cell population having a spread in cycle time (and in lengths of the component phases) the phase boundaries in the diagram for P-cells are not vertical but are as shown in the lower half of Fig. 9 (Barrett, 1966*a*).

FIG. 9.—Age distribution diagrams calculated for the BICR/A2 tumour. The upper diagram is obtained by ignoring spread in cell cycle time ; the lower diagram is the corresponding curve when spread is taken into account.

*Analysis of the continuous labelling curve.*

The method of analysis was to assume the distribution of cell cycle times obtained from the labelled mitoses data, to draw the corresponding age distribution diagrams first for Model A and then for Model B with selected values for the constant $\alpha$, and for each of these to calculate a continuous labelling curve, seeking one which would fit the experimental data closely. In these calculations, it was first assumed that the spread in $G_2$ would have a negligible effect on the continuous labelling curve and could therefore be assumed to be zero. This assumption is reasonable, since its effect is only on curvature, and then only over the first 3

or 4 hours of continuous labelling. When this assumption is made, the labelling index under continuous labelling first increases until at the end of the $G_2$ period it is given by the fractional area of the age distribution diagram occupied by cells in the S and $G_2$ periods. Thereafter, labelled cells begin to appear at the beginning of the diagram and the boundary between labelled and unlabelled cells (a vertical line) sweeps through the $G_1$ compartment until all P-cells are labelled. At the same time (on Model B) the vertical line sweeps through the Q-cell compartment also and at any point the labelling index may be calculated by determining the appropriate fractional area.

In the case of the BICR/M1 tumour, calculation on the basis of Model A gives a reasonable fit for the first 12 hours but thereafter the theoretical curve reaches 100% much too quickly. The curve in Fig. 6 is calculated on Model B for $\alpha = 1{\cdot}95$, i.e., on the assumption that the Proliferative Fraction was 95% (in principle, when $T_c$ and $T_d$ are known, $\alpha$ is specified by equations (iii) and (iv) above, but in fact a value of $\alpha = 1{\cdot}95$ is entirely consistent with the measured values of $T_c$ and $T_d$ for BICR/M1). One is not justified in moving on to Model C in this case ; the experimental results are satisfactorily explained by a model in which the distribution of cycle times is that given in Fig. 8 and in which there are about 5% non-proliferating cells. Although the limits of error on this figure are fairly large it is nevertheless clear that their proportion could not be more than about 10%.

In the case of the BICR/A2 tumour, Model A gives an impossible result, since it predicts almost 100% labelling at 70 hours. Fig. 7 shows the theoretical curve (a) calculated on Model B, $\alpha$ being given by equation (iii) above as 1·26 (Proliferative Fraction of 26%). This curve is in satisfactory agreement with the initial labelling index and the experimental points out to 36 hours, but thereafter it does not rise sufficiently quickly.

We have examined two alternative explanations of this discrepancy : first that it results from the presence within the tumour of cells with long cycle times, cells which form an extended " tail " on the cycle time distribution and which in our analysis have been included in the Q-cell compartment ; secondly, that the discrepancy is due to cell loss from the tumour. As regards the first of these possibilities, it is probably true that the firm distinction which we have made in the definition of Model B between P-cells and Q-cells is an over-simplification of the actual situation. It is also true that the presence of cells with cycle times in the region of 100 hours would help to raise the theoretical continuous labelling curve at later intervals. It is difficult, however, to see how this factor could completely resolve the discrepancy. In Fig. 7 the discrepancy in labelling index at 100 hours amounts to 20% of all cells. To resolve this, a number of Q-cells equal to at least 20% of the whole cell population would have to be assumed proliferative and since only those cells which label before 100 hours would affect discrepancy, their cycle times could not be much in excess of this time. The Proliferative Fraction as already found is, however, 26% and hence this assumption involves almost doubling the Proliferative Fraction by cells having up to twice the mean cycle time. The difficulty of assuming such a model is that the corresponding labelled mitoses curve would differ considerably from that which has so far been computed. Despite the fact that the labelled mitoses data for BICR/A2 show relatively large scatter, they nevertheless eliminate the possibility that the " tail " of the cell cycle time distribution could be so large as to double the proportion of proliferative cells, hence it would seem that this explanation can only partly resolve the discrepancy.

On the other hand, there is no doubt that the assumption of some degree of cell loss could explain the observed discrepancy. Since what is required here is a steepening of the curve with minimum effect on the early points, cell loss of type (i) (see definition of Model C above) might seem to be the best choice. By contrast the other two types of cell loss involve the loss of some labelled cells after relatively short intervals. Fig. 7 shows the effect of assuming that Q-cells have a limited life-span either of 1 or $1\frac{1}{2}$ times the tumour doubling time (190 or 285 hours); the corresponding Proliferative Fractions are 39% and 33% respectively. These assumptions, which do improve the fit to the experimental points, imply that respectively 18 or 12 cells were lost for every 100 produced at mitosis.

Comparing these two explanations for the observed discrepancy between the theoretical and experimental continuous labelling curves, it would seem likely that both may contribute to some extent. It is difficult to avoid the conclusion that some cell loss was taking place from the BICR/A2 tumour, but its extent is difficult to assess because the importance of an extended tail on the cycle time distribution cannot be judged from the present data.

### CONCLUSIONS

The two tumours examined in the present investigation had volume doubling times which differed by a factor of just over 8. This factor was not, however, reflected in a similar difference in mean cycle time. If the assumption is correct, as seems to be borne out by the tritiated thymidine results, that the cell populations behaved in each case as though they consisted of two groups of cells, one group proliferating with a specified distribution of cell cycle times and the other group non-proliferating, then the mean cell cycle times seem to be in the ratio of 2·6 : 1. The difference in growth rate is therefore largely the result of the proportion of proliferating cells being about 95% in the case of BICR/M1 and only about 30% in the case of BICR/A2.

The results have also enabled some, though rather imprecise, estimates to be made of the degree of cell loss from the two tumours. BICR/M1, the more rapidly proliferating, which had been maintained by repeated transplantation for 10 years, had no detectable cell loss; for BICR/A2, which was only in its 4th transplant, the loss of up to 20 cells for every 100 produced at mitosis, would be consistent with the data.

We gratefully acknowledge the support which Professor L. F. Lamerton has given to this work and the inspiration which we have gained from him. We also wish to thank Miss J. Lambert, Mrs. B. I. Lord and Mrs. R. Tarbutt for their assistance in autoradiography and histology.

### REFERENCES

BARRETT, J. C.—(1966*a*) Ph.D. Thesis, University of London.—(1966*b*) *J. natn. Cancer Inst.* **37**, 443.
BASERGA, R., KISIELESKI, W. E. AND HALVORSEN, K.—(1960) *Cancer Res.*, **20**, 910.
BERTALANFFY, F. D. AND McASKILL, C.—(1964*a*) *Oncologia*, **18**, 120.—(1964*b*) *J. natn. Cancer Inst.*, **32**, 535.

Blum, H. F.—(1943) *J. natn. Cancer Inst.*, **4**, 21.
Brues, A. M., Weiner, A. E. and Andervont, H. B.—(1939) *Proc. Soc. exp. Biol. Med.*, **42**, 374.
Cairnie, A. B., Lamerton, L. F. and Steel, G. G.—(1965) *Expl Cell Res.*, **39**, 539.
Hunter, J. C.—(1955) *J. natn. Cancer Inst.*, **16**, 405.
Johnson, H. A.—(1961) *Cytologia*, **26**, 32.
Kim, J. H. and Evans, T. C.—(1964) *Radiat. Res.*, **21**, 129.
Laird, A. K.—(1964) *Br. J. Cancer*, **28**, 490
Lord, B. I.—(1963) *J. photogr. Sci.*, **11**, 342.
Mayneord, W. V.—(1932) *Am. J. Cancer*, **16**, 841.
McCredie, J. A., Inch, W. R., Kruuv, J. and Watson, T. A.—(1965) *Growth*, **29**, 331
Mendelsohn, M. L.—(1962*a*) *J. natn. Cancer Inst.*, **28**, 1015.—(1962*b*) *Science, N.Y.*, **135**, 213.—(1965) in "Cellular radiation biology", University of Texas. (Williams and Wilkins).
Mendelsohn, M. L., Dohan, F. C. and Moore, H. A.—(1960) *J. natn. Cancer Inst.*, **25**, 477.
Mottram, J. C.—(1935) *J. Path. Bact.*, **40**, 407.
Quastler, H.—(1963) "Cell Proliferation" edited by Lamerton, L. F. and Fry, R. J. M. Oxford (Blackwell).
Quastler, H. and Sherman, F. G.—(1959) *Expl Cell Res.*, **17**, 420.
Schreck, R.—(1935) *Am. J. Cancer*, **24**, 807.
Steel, G. G.—(1966) *Nature, Lond.*, **210**, 806.
Steel, G. G., and Lamerton, L. F.—(1966) *Br. J. Cancer*, **20**, 74.
Wimber, D. E.—(1963) "Cell Proliferation" edited by Lamerton, L. F. and Fry, R. J. M. Oxford (Blackwell).